# Características epidemiológicas de pacientes pediátricos con candidiasis invasora en una unidad de cuidados intensivos del suroccidente colombiano

**DOI:** 10.7705/biomedica.7444

**Published:** 2025-03-28

**Authors:** Paola Marsela Pérez-Camacho, Carmen Vargas-Morán, Laura Torres-Canchala, Camila Ariza-Insignares, Lina M. Sandoval-Calle, Inés Elvira Gómez-Hemández, Paula Solís-Núñez, Juliana V. Cedeño-Castaño, Ana M. Aguilar-González, Jaime Alberto Patiño-Niño

**Affiliations:** 1 Servicio de Infectología Pediátrica, Fundación Valle del Lili, Cali, Colombia Fundación Valle del Lili Fundación Valle del Lili Cali Colombia; 2 Facultad de Ciencias de la Salud, Universidad Icesi, Cali, Colombia Universidad Icesi Universidad Icesi Cali Colombia; 3 Centro de Investigaciones Clínicas, Fundación Valle del Lili, Cali, Colombia Universidad Icesi Fundación Valle del Lili Cali Colombia

**Keywords:** Candida, candidiasis, candidemia, candidiasis invasiva, unidades de cuidados intensivos pediátricos, pediatría, Candida, candidiasis, candidemia, candidiasis, invasive, intensive care units, pediatric, pediatrics

## Abstract

**Introducción.:**

Las especies de *Candida* son el principal agente etiológico de infecciones fúngicas en la población pediátrica, especialmente en neonatos y pacientes atendidos en la unidad de cuidados intensivos. La candidiasis invasiva se relaciona con desenlaces clínicos desfavorables como tiempo de hospitalización prolongado y mortalidad.

**Objetivo.:**

Describir las características demográficas, clínicas y microbiológicas de los pacientes pediátricos (mayores de un mes y menores de 18 años) hospitalizados en una unidad de cuidados intensivos pediátricos con diagnóstico de candidiasis invasora entre el 2012 y el 2020.

**Materiales y métodos.:**

Se adelantó un estudio observacional, retrospectivo, de tipo cohorte, en un centro de alta complejidad del suroccidente colombiano.

**Resultados.:**

Se incluyeron 100 pacientes pediátricos con diagnósticos de candidiasis invasora, el 51 % de sexo femenino con una mediana de edad de 6,5 años (RIC = 2-11,5). Se obtuvieron 114 aislamientos de *Candida* spp. La mediana de estancia hospitalaria fue de 51 días (RIC = 29-77) y de estancia en la unidad de cuidados intensivos pediátricos de 27 días (RIC = 16-58). El 85 % de los pacientes presentó taquicardia 24 horas antes del aislamiento de *Candida* spp. El 49,1 % de los aislamientos se encontró en las muestras de sangre (49,1 %), el lavado broncoalveolar (21,9 %) y el líquido peritoneal (20,2 %). La especie aislada más frecuente fue *Candida albicans* (36,8 %), seguida de *C. parapsilosis* (22,8 %) y *C. tropicalis* (21,1 %). El 36 % de los pacientes falleció.

**Conclusiones.:**

La candidiasis invasora es una entidad común en las unidades de cuidados intensivos pediátricos y es un desafío por su alta morbilidad, prolongación de la estancia hospitalaria y tasa de mortalidad elevada. Aunque *C. albicans* sigue siendo la especie predominante, las especies de *C*. no *albicans* han mostrado una tendencia creciente, lo que plantea nuevos retos diagnósticos y terapéuticos.

## Abstract (en)

**Introduction.:**

*Candida* species are the main etiological agent of fungal infections in the pediatric population, especially in neonates and in intensive care unit patients. Invasive candidiasis is associated with unfavorable clinical outcomes, such as prolonged hospitalization time, and mortality.

**Objective.:**

To describe demographic, clinical and microbiological characteristics of pediatric patients (older than one month and younger than 18 years) hospitalized in a pediatric intensive care unit with a diagnosis of invasive candidiasis between 2012 and 2020.

**Materials and methods.:**

A retrospective, observational, cohort study was conducted at a high-complexity center in southwestern Colombia.

**Results.:**

We included 100 pediatric patients diagnosed with invasive candidiasis, 51% female with a median age of 6.5 years (IQR = 2-11.5). We obtained 114 isolates of *Candida* spp. The median hospital stay was 51 days (IQR = 29-77), with a pediatric intensive care unit stay of 27 days (IQR = 16-58). Tachycardia was present in 85% of the patients 24 hours before *Candida* spp. isolation. Nearly half of the isolates were found in bloodstream samples (49.1%), respiratory samples (21.9%), and peritoneal fluid (20.2%). The most frequently isolated species were *C. albicans* (36.8%), followed by non-albicans species, such as *C. parapsilosis* (22.8%), and *C*. *tropicalis* (21.1%). The overall mortality rate at discharge was 36%.

**Conclusions.:**

In pediatric intensive care units, invasive candidiasis is a common condition representing a significant threat due to its high morbidity, prolonged hospital stay, and considerable mortality rate. While *C. albicans* remains as the predominant species, non-*albicans Candida* species exhibit a growing trend, posing new diagnostic and therapeutic challenges.

La Organización Mundial de la Salud reportó que en el 2022 fallecieron 2,3 millones de neonatos en el mundo. Las infecciones son la tercera causa de muerte más frecuente, después de las complicaciones clínicas asociadas con la prematuridad y el parto. La mayoría de las infecciones neonatales son causadas por especies de *Candida* y las tasas de infección varían según la ubicación geográfica, las características clínicas que aumentan la susceptibilidad de los pacientes y las prácticas de asepsia y antisepsia de los servicios de salud ^1^^-^^4^. Por ejemplo, los pacientes inmunocomprometidos o aquellos que requieren catéter venoso central o sonda urinaria presentan infecciones por *Candida* spp. del torrente sanguíneo con una prevalencia del 10 al 20 % ^5^^-^^7^.

La infección invasora por *Candida* spp., conocida también como candidiasis invasora, incluye la presencia de levaduras en la sangre (candidemia) y la diseminación del hongo a órganos profundos, con candidemia asociada o sin ella ^8^. A nivel mundial, se reportan aproximadamente 700.000 casos anuales de candidiasis invasora ^4^. En los países de altos ingresos, la tasa bruta de mortalidad por candidiasis invasora se estima entre el 12 y el 37 %, mientras que en los países de bajos y medianos ingresos, la mortalidad varía entre el 8,9 y el 75 %. En la población pediátrica, las infecciones por especies de *Candida* son la tercera causa de infecciones sanguíneas en unidades de cuidados intensivos pediátricos y neonatales ^6^^,^^7^^,^^9^^,^^10^. Además, estas infecciones están asociadas con una mayor estancia hospitalaria -estimada en un promedio de 21 días adicionales a lo que la enfermedad de base requiere-, lo que también incrementa los costos hospitalarios ^11^^,^^12^.

El desarrollo de candidiasis invasora está asociado con múltiples factores de riesgo, que se pueden clasificar en dos categorías principales: aquellos relacionados con el huésped (como diagnósticos de malignidad o inmunosupresión, neutropenia y edad) y aquellos relacionados con la atención médica (medidas de asepsia y antisepsia, uso de catéter venoso central o accesos sanguíneos, nutrición parenteral, uso prolongado de antibióticos intravenosos, corticoesteroides, administración previa de antifúngicos o cirugías recientes, especialmente abdominales) ^7^^,^^9^^,^^11^^-^^13^. En los países de altos ingresos, la mortalidad por candidiasis invasora es particularmente alta en los neonatos con un peso inferior a los 1.000 g o una edad gestacional menor de 28 semanas. En contraste, en los países de bajos y medianos ingresos, la candidiasis invasora afecta a una población más amplia y se ha observado un aumento en la incidencia de especies de *Candida* no *albicans*, como *C. parapsilosis*, *C. krusei* y *C.auris*, las cuales presentan menor sensibilidad al fluconazol ^3^.

En la última década, se ha reportado un cambio en la incidencia de candidiasis invasora y en la distribución de especies. Por ejemplo, los *Centers for Disease Control and Prevention* (CDC) reportaron una disminución de la candidemia en niños mayores de cinco años en Estados Unidos, de 1,8 casos por 100.000 nacidos en el 2009 a 0,8 casos por 100.000 nacidos en el 2014 ^14^. Como se mencionó, aunque *C. albicans* sigue siendo la especie más aislada, la incidencia de las especies no albicans ha aumentado (7,11,15). Esto es relevante porque algunas especies de *C*. no *albicans*, como *C. parapsilosis* y *C. tropicalis*, presentan patrones de resistencia a antifúngicos, lo que genera un desafío terapéutico ^10^.

En Latinoamérica, diversos estudios han abordado la candidiasis en la población neonatal, pero las demás edades pediátricas no suelen ser comúnmente incluidas a pesar de su relevancia clínica. Nucci *et al*. describieron que el 44,2 % de los casos de candidiasis invasora -en los 21 hospitales latinoamericanos evaluados- correspondía a niños mayores de un mes y menores de 18 años. La especie más comúnmente aislada fue *C. albicans*, presente en el 37,2 % de los individuos estudiados, seguida de *C. parapsilosis* (26,5 %) y *C. tropicalis* (17,6 %) (16). En el 2021, México reportó una incidencia de candidiasis invasora de 2,27 por cada 1.000 recién nacidos vivos, y *C. albicans* fue la especie más prevalente (35,3 %). También se documentaron factores de riesgo asociados a candidiasis invasora como: necesidad de respiración mecánica asistida (OR = 3,04; IC _95 %_: 1,13-8,14), terapia antibiótica sistémica (OR = 7,48; IC _95 %_: 1,30-42,9), el número de esquemas antimicrobianos (OR = 2,02; IC _95 %_: 1,01-4,03) y los días con nutrición parenteral total (OR = 1,14; IC _95 %_: 1,04-1,25) o con catéter venoso central (OR = 1,11; IC_95 %_: 1,02-1,20) ^17^.

En 2012, en Colombia se estimó una incidencia de candidemia de 1,98 casos por cada 1.000 hospitalizaciones ^18^. Sin embargo, dicha cifra puede variar dependiendo del área geográfica y del servicio de hospitalización que esté tratando al paciente. Varios estudios del país han evidenciado una disminución de la frecuencia relativa de *C. albicans* y un aumento de otras especies como *C. parapsilosis* y *C. glabrata*, lo cual tiene implicaciones en el manejo farmacológico debido a su menor sensibilidad al fluconazol ^18^.

El diagnóstico de candidiasis invasora es un reto para el personal de salud, ya que los cultivos de sangre son poco sensibles y específicos para la detección de *Candida* spp. Por ello, se ha implementado el uso de otros métodos diagnósticos, como el sistema T2Candida™ Panel, la amplificación de ácidos nucleicos y la detección enzimática de glucano ^14^. Además, el tratamiento de las infecciones fúngicas invasivas sigue siendo un desafío, especialmente por la resistencia a los antifúngicos, lo que ha llevado a la incorporación y el estudio de medicamentos como la anfotericina B liposómica, la micafungina y la anidulafungina en la población pediátrica y neonatal ^19^^-^^22^.

Dada la importancia clínica y epidemiológica de la candidiasis invasora y la escasa información publicada hasta el momento en población pediátrica (diferente al periodo neonatal), el objetivo de este estudio fue describir las características demográficas y clínicas, y la mortalidad de pacientes pediátricos con diagnóstico de candidiasis invasora. La población estudiada estuvo hospitalizada en una unidad de cuidados intensivos pediátricos de una institución de alta complejidad del suroccidente colombiano entre el 2012 y el 2020.

## Materiales y métodos

Se llevó a cabo un estudio descriptivo y retrospectivo en el que se recolectaron datos de las historias clínicas del sistema de registro de la Fundación Valle del Lili de *Systems, Applications & Products in Data Processing*, SAP. Con ayuda del Departamento de Estadística y del Comité Institucional de Infecciones, se hizo la búsqueda de los pacientes pediátricos con aislamiento microbiológico de *Candida* spp. a partir de muestras estériles (hemocultivos, líquido peritoneal, cefalorraquídeo o de lavado broncoalveolar), hospitalizados en la unidad de cuidados intensivos pediátricos incluida la de cuidados cardiovasculares entre enero del 2012 y diciembre del 2020. En este periodo se atendieron 8.394 pacientes en la unidad de cuidados intensivos pediátricos y 2.513 en la de cuidados cardiovasculares, para un total de 10.907 pacientes pediátricos. En promedio, a cada unidad de cuidados intensivos ingresaron 1.211 pacientes por año.

Se excluyeron los pacientes menores de 30 días (un mes) o mayores de 18 años, hospitalizados en la unidad de cuidados intensivos neonatales o con ingresos previos en esta unidad, con aislamientos asociados a infecciones de las vías urinarias o aislamientos microbiológicos de especies de *Candida* de muestras no estériles y estancia hospitalaria menor de siete días.

Las muestras fueron cultivadas en el laboratorio de la institución, en medios selectivos como agar Sabouraud dextrosa y agar cromogénico para levaduras. Las placas se incubaron a 35-37 °C durante 48 a 72 horas. Posteriormente, se hizo la identificación de las especies de *Candida* mediante la observación de las características morfológicas y de su color en el agar cromogénico. Mediante el sistema Vitek 2™ se determinó la identificación microbiana y la prueba de sensibilidad antimicrobiana de forma automatizada. Se calculó la concentración inhibitoria mínima para cada antifúngico según los manuales M27M44S y M57S del *Clinical and Laboratory Standards Institute* (CLSI). La cepa se consideró sensible (S) cuando la concentración inhibitoria mínima era igual o menor del punto de corte establecido, intermedia (I) cuando dicho valor se encontraba en un rango en el cual el éxito del tratamiento era incierto, y resistente (R) cuando superaba el punto de corte.

Entre las variables del estudio se incluyeron características epidemiológicas (edad y sexo); clínicas (signos y síntomas, antecedentes de importancia, sitio de la infección, diagnósticos de sepsis grave o choque séptico, según los criterios diagnósticos propuestos por la Conferencia Internacional de Consenso sobre Sepsis Pediátrica del 2005 ^23^); motivo de ingreso a la unidad de cuidados intensivos pediátricos o a la unidad cardiovascular, y microbiológicas (identificación de la especie de *Candida* y el antifungigrama cuando estaba disponible). Además, se incluyeron variables que describen factores de riesgo reconocidos como: tiempo de estancia hospitalaria, tratamientos antibióticos previos, uso de catéteres intravasculares, cirugía abdominal, criterios para sepsis grave y características microbiológicas del aislamiento de las especies de *Candida*, incluyendo el patrón de sensibilidad, en los casos donde se reportaba.

Las variables continuas se representaron con medianas y rangos intercuartílicos (RIC) y las variables categóricas, con tablas de frecuencias y porcentajes. El análisis se realizó con el paquete estadístico Stata™, versión 14.0 (Stata Corp., TX). Esta investigación se acogió a las normas internacionales sobre investigación biomédica, la Declaración de Helsinki y del *Council for International Organizations of Medical Sciences* (CIOMS). Dado que los datos se obtuvieron para la práctica clínica habitual, no se exigió el consentimiento informado. Este estudio fue aprobado por el Comité en Investigación Biomédica de la Fundación Valle del Lili, Cali, Colombia (Protocolo No. 1277).

## Resultados

Se identificaron 498 pacientes con infecciones por *Candida* spp. según la Clasificación Internacional de Enfermedades (códigos CIE-10). Al aplicar los criterios de inclusión del estudio, se obtuvieron 100 pacientes pediátricos con diagnósticos de candidiasis invasora de quienes se obtuvieron 114 aislamientos de *Candida* spp.

La mediana de edad fue de 6,5 años (RIC = 2-11,5) y no se identificó una diferencia significativa en la distribución por sexo. El 76 % de los pacientes tenía una enfermedad de base al momento de su ingreso a la unidad de cuidados intensivos pediátricos o a la cardiovascular, siendo las cardiopatías (35,5 %) las más frecuentes. No hubo ningún paciente que tuviera como antecedente trasplante de médula ósea. La mediana de estancia hospitalaria fue de 51 días (RIC = 29-7) y en unidad de cuidados intensivos fue de 27 días (RIC = 16-8). Se registró una mortalidad general del 36 % (cuadro 1).

**Cuadro 1:** Características demográficas y clínicas de pacientes con diagnóstico de candidiasis invasiva (N = 100)

		n	%
Sexo			
	Femenino	51	51,0
	Masculino	49	49,0
Edad (años), mediana		6.5	(2-11.5)
Procedencia del paciente			
	Urgencias	25	25,0
	Hospitalización	30	30,0
	Unidad de cuidados intensivos pediátricos de otra institución	7	7,0
	Otra institución	38	38,0
	Enfermedad de base	76	100
	Cardiopatía	27	35,5
	Patología neurológica	19	25,0
	Trastorno hepático	17	22,4
	Enfermedad gastrointestinal	14	18,4
	Trastorno hematooncológico	10	13,2
	Patología metabólica	10	13,2
	Nefropatía	9	11,8
	Trasplante hepático	8	10,5
	Patología respiratoria	6	7,9
	Inmunodeficiencia	5	6,6
	Patología autoinmunitaria	5	6,6
	Trasplante renal	2	2,6
	Trastorno musculoesquelético	1	1,3
	Trasplante de médula ósea	0	0,0
Días de hospitalización**		51	(29-77)
Días en UCI, mediana**		27	(16-58)
Razón de ingreso a la unidad de cuidados intensivos			
	Infección asociada a los cuidados de la salud	30	30,0
	Infección adquirida en la comunidad	23	23,0
	Cirugía	24	24,0
	Cardiopatía	18	18,0
	Trasplante hepático	10	10,0
	Trauma	8	8,0
	Enfermedad hematooncológica	5	5,0
	Tumores sólidos	2	2,0
	Trasplante renal	2	2,0
	Trasplante de médula ósea	0	0,0
Muerte al egreso		36	36,0

* Mediana (RIC)

En cuanto a los datos de laboratorio, durante el episodio de candidiasis invasora, el 11 % (n = 11) de los pacientes desarrolló neutropenia por más de 10 días. De estos, 4 pacientes tenían enfermedad hematooncológica de base (3 padecían leucemia linfoblástica aguda y 1 tenía aplasia medular) y presentaron una mediana de 1.010 neutrófilos en el día uno de la neutropenia (RIC = 270-1.380) y de 80 neutrófilos en el día 10 de la neutropenia (RIC = 10-960).

En cuanto a la presencia de factores de riesgo asociados al desarrollo de candidiasis invasora, se encontró que 15 días antes del diagnóstico el 88 % de los pacientes había tenido un catéter venoso central o había sido sometido a otros procedimientos invasivos como respiración mecánica asistida (65 %), nutrición parenteral (58 %) o transfusiones sanguíneas (60 %). Además, entre los pacientes que habían sido intervenidos quirúrgicamente (n = 46), en más de la mitad, la cirugía fue abdominal (n = 25). Se evidenció que el uso de antibióticos, corticoesteroides e inhibidores de la bomba de protones tenía una asociación con el desarrollo de candidiasis invasora (cuadro 2).

**Cuadro 2:** Factores de riesgo documentados 15 días antes de la infección por *Candida* spp. (N = 100)

		n	%
Uso de antibióticos dos semanas antes del diagnóstico		95	95,0
Uso de catéter venoso central		88	88,0
Uso de inhibidores de la bomba de protones o bloqueadores de H^2^		67	67,0
Respiración mecánica invasiva		65	65,0
Uso de catéteres vesicales		63	63,0
Infección documentada los últimos 15 días*		61	61,0
Trasfusiones de hemoderivados		60	60,0
Uso de nutrición parenteral		58	58,0
Algún tipo de cirugía		46	46,0
	Abdominal	25	25,0
	Cardiovascular	15	15,0
	Ortopédica	4	4,0
	Oftalmológica	2	2,0
Uso de corticoesteroides		44	44,0
Uso de antifúngicos		26	26,0
Bacteriemia durante el uso de antifúngicos		9	9,0
Días de antibiótico antes del diagnóstico**		9	(6-14)
	Vancomicina	55	55,0
	Meropenem	49	49,0
	Piperacilina-tazobactam	49	49,0
	Cefalosporina	17	17,0
	Cefepime	17	17,0
Hemodiálisis		8	8,0
Días de nutrición parenteral**		7	(4-16)
Días de uso de corticoesteroides**		6	(3-10)
Días de uso de antifúngicos**		5	(3-14)
Finalidad del uso del antifúngico		5	5,0
	Profilaxis	19	19,0
	Tratamiento	2	2,0
	Sin datos		12,0
Tipo de antifúngico recibido		12	12,0
	Fluconazol	12	4,0
	Caspofungina	4	4,0
	Anfotericina B	4	
	Voriconazol		

* Se incluyeron todas las infecciones previas de cualquier etiología.

** Mediana (RIC)

Veinticuatro horas antes del diagnóstico de candidiasis se recolectaron datos de los parámetros clínicos y paraclínicos que hacen parte de los criterios de sepsis grave (cuadro 3). La taquicardia fue el signo más común (87 %) junto con la fiebre (51 %). En cuanto a las alteraciones de laboratorio, el 65,6 % de los pacientes presentó alguna anomalía en el conteo de leucocitos (leucopenia y leucocitosis). También se evaluó la presencia de disfunciones orgánicas: disfunción cardiovascular (definida como la presencia de hipotensión resistente a la reanimación hídrica) en el 22 % y necesidad de vasoactivos en el 42 %; disfunción respiratoria por la necesidad de respiración mecánica invasiva y no invasiva (76 %) y necesidad de una fracción inspirada de oxígeno (FiO_2_) mayor del 50 % (55 %). Con relación a la disfunción neurológica, la escala de Glasgow fue menor de 11 puntos en 7 pacientes. Sin embargo, no fue posible recolectar este dato en todos los casos, ya que la mayoría requirió respiración mecánica. Este procedimiento implica sedación y analgesia y, por lo tanto, no permite una valoración neurológica adecuada.

**Cuadro 3:** Criterios de sepsis grave 24 horas antes del diagnóstico confirmado de *Candida* spp., según la Conferencia Internacional de Consenso sobre Sepsis Pediátrica del 2005

	n	%
Temperatura mayor de 38 °C o menor de 36 °C	51	51,0
Taquicardia	87	87,0
Taquipnea	76	76,0
Número de leucocitos en el momento de la toma del cultivo	10.490	(7.090 a 16.250)
Hipotensión después de reanimación hídrica	22	22,0
Necesidad de vasoactivos	42	42,0
Base exceso < -5	27	27,0
Ácido láctico > 2	19	19,0
Oliguria	14	14,0
Llenado capilar > 5 s	10	10,0
INR (International Normalized Ratio) > 2	6	6,0
Número de plaquetas en el momento de la toma del cultivo	122.000	(46.000 a 226.500)
Niveles de alanina aminotransferasa**	27,2	(13,4 a 57)
Necesidad de respiración mecánica invasiva o no invasiva	76	76,0
Oxigeno suplementario con FiO2 > 50 %	55	55,0
Glasgow < 11	7	7,0
Primera creatinina tomada después del ingreso**	0,37	(0,25 a 0,67) mg/dl
Creatinina más alta durante la hospitalización**	0,34	(0,24 a 0,57) md/dl

* Plaquetas < 150.000 por ml

** Mediana (RIC)

En cuanto a las características microbiológicas de los aislamientos (n = 114), casi la mitad de los que resultaron positivos se obtuvieron de muestras de sangre (49,1 %), seguidas del lavado broncoalveolar (21,9 %) y del líquido peritoneal (20,2 %). Se identificó que la especie aislada más frecuente fue *C. albicans* (36,8 %), seguida por especies de *C*. no *albicans* como *C. parapsilosis* (22,8 %) y *C. tropicalis* (21,1 %) (cuadro 4).

**Cuadro 4:** Características microbiológicas de aislamientos positivos para *Candida* spp. (N = 114)

Tipo de muestra		n	%
Sangre		56	49,1
	Respiratoria	25	21,9
	Líquido peritoneal	23	20,2
	Líquido pleural	2	1,8
	Otros*	8	7,0
Especies aisladas			
	*Candida albicans*	42	36,8
	*Candida parapsilosis*	26	22,8
	*Candida tropicalis*	24	21,1
	*Candida glabrata*	8	7,0
	*Candida lusitaniae*	6	5,3
	*Candida guilliermondii*	4	3,5
	*Candida auris*	2	1,8
	*Candida krusei*	1	0,9
	*Candida intermedia*	1	0,9

* otros: Colección intrabdominal, secreción mediastinal

De los 114 aislamientos positivos, 14 correspondieron a episodios con segundos aislamientos de *Candida* spp.: 8 fueron coinfecciones (dos especies diferentes de *Candida* en el mismo cultivo) y los 6 restantes fueron cultivos positivos para especies de *Candida* en dos tiempos diferentes durante la misma hospitalización (cuadro 5).

**Cuadro 5:** Características microbiológicas de los segundos aislamientos de *Candida* spp. (n = 14)

Lugar del primer aislamiento		**Especie de *Candida***	Fecha (DD.MM.AA)	Lugar del segundo aislamiento	Especie	Fecha (DD.MM.AA)	Tipo del segundo aislamiento
Líquido peritoneal		*C. krusei*	06.01.17	Líquido peritoneal	*C. tropicalis*	06.01.17	Coinfección
Vías respiratorias		*C. albicans*	14.09.15	Lavado broncoalveolar	*C. tropicalis*	14.09.15	Coinfección
Líquido peritoneal		*C. albicans*	30.03.18	Sangre	*C. guilliermondii*	13.04.18	Segunda infección
Sangre		*C. glabrata*	13.06.21	Lavado broncoalveolar	*C. tropicalis; C. glabrata*	13.06.21	Coinfección
	Vías respiratorias	*C. albicans*	14.08.14	Lavado broncoalveolar	*C. tropicalis*	14.08.14	Coinfección
	Líquido peritoneal	*C. parapsilosis*	26.03.16	Líquido peritoneal	*C. tropicalis*	26.03.16	Coinfección
	Líquido peritoneal	*C. albicans*	05.06.16	Líquido peritoneal	*C. parapsilosis*	05.06.21	Coinfección
	Sangre	*C. auris*	09.03.18	Secreción del mediastino	*C. auris*	20.03.18	Segunda infección
	Sangre	*C. glabrata*	03.08.15	Líquido peritoneal	*C. albicans*	03.08.15	Coinfección
Líquido peritoneal		*C. tropicalis*	19.02.17	Líquido peritoneal	*C. albicans*	19.02.17	Coinfección
Sangre		*C. parapsilosis*	05.02.12	Sangre	*C. albicans*	29.02.12	Segunda infección
Vías respiratorias		*C. tropicalis*	27.11.12	Sangre	*C. parapsilosis*	13.12.12	Segunda infección
Colección suprahepática		*C. albicans*	25.09.12	Absceso peritoneal	*C. parapsilosis*	26.12.12	Segunda infección
Líquido peritoneal		*C. glabrata*	29.07.16	Sangre	*C. glabrata*	22.08.16	Segunda infección

El patrón de sensibilidad y resistencia a los antifúngicos de las especies identificadas en todos los aislamientos (n = 114) se resume en el cuadro 6. Es importante aclarar que no en todos los casos se realizaron pruebas de sensibilidad para todos los antifúngicos: el 62 % (71/114) fue evaluado para fluconazol, el 50,8 % (58/114) para voriconazol, el 9,6 % (11/114) para anfotericina B y el 55 % (63/114) para caspofungina.

**Cuadro 6:** Antifungigrama según la especie de *Candida* de los primeros y segundos aislamientos

Antifúngico*		Fluconazol (n = 71)				Voriconazol (n = 58)				Anfotericina B (n = 11)				Caspofungina (n = 63)		
Especie de Candida**	S	I	R	Sd	S	I	R	Sd	S	I	R	Sd	S	I	R	Sd
*Candida albicans* (n = 82)	26	0	1	15	28	0	0	14	0	0	0	42	27	0	0	15
*Candida parapsilosis* (n = 40)	3	0	14	9	8	1	0	17	0	0	2	24	12	0	0	14
*Candida tropicalis* (n = 43)	14	0	1	9	14	0	0	10	0	0	0	24	14	0	0	10
*Candida glabrata* (n = 16)	2	3	2	1	2	0	0	6	2	0	0	6	3	2	0	3
*Candida lusitaniae* (n = 11)	2	0	0	4	2	0	0	4	2	0	3	1	2	0	0	4
*Candida guilliermondii* (n = 6)	2	0	0	2	2	0	0	2	0	0	0	4	2	0	0	2
*Candida auris* (n = 4)	0	0	1	1	1	0	0	1	0	0	1	1	1	0	0	1
*Candida krusei* (n = 1)	0	0	0	1	0	0	0	1	0	0	1	0	0	0	0	1
*Candida Intermedia* (n = 0)	0	0	0	1	0	0	0	1	0	0	0	1	0	0	0	1

S: sensible, I: intermedio, R: resistente, Sd: sin datos (No se hizo antifungigrama.)

* Se señala el número de aislamientos por columna para cada antifúngicos utilizado en los antifungigramas.

** Se señala el número de aislamientos por fila por especie de Candida evaluada en los antifungigramas.

Durante el periodo del estudio se documentó la muerte de 36 pacientes: 27 murieron en los 30 días posteriores al diagnóstico de candidiasis y, de estos, 16 se atribuyeron a la infección por *Candida* spp. según criterio médico. De los 36 fallecidos, el 44,4 % (n = 16) tuvo infección por *C. albicans*, el 22,2 % (n = 8) por *C. parapsilosis*, el 22,2 % (n = 8) por *C. tropicalis* y el 11,1 % (n = 4) por *C. glabrata*. Solamente uno de estos pacientes tuvo una coinfección, confirmada por el aislamiento de *C. glabrata* en el hemocultivo y de *C. glabrata* y *C. tropicalis* en la muestra de lavado broncoalveolar.

## Discusión

Este es el primer estudio de candidiasis invasora en Colombia de pacientes pediátricos hospitalizados en la unidad de cuidados intensivos pediátricos o en la cardiovascular, en el cual se describen las características demográficas, clínicas y microbiológicas de 100 pacientes con diagnóstico de candidiasis invasora del 2012 al 2020.

Las especies de *Candida* son el principal agente causal de las infecciones fúngicas invasoras en las unidades de cuidados intensivos pediátricos o de adultos. En el presente estudio, se encontró que el aislamiento de *Candida* spp. fue más común en las muestras de sangre (49,1 %), el lavado broncoalveolar (21,9 %) y el líquido peritoneal (20,2 %). Esto se asemeja a los resultados de un estudio multicéntrico realizado en Turquía, en el que se incluyeron pacientes pediátricos de todos los servicios (no exclusivamente de la unidad de cuidados intensivos pediátricos), y reportaron que cerca del 51 % de los aislamientos fueron de casos de candidemia versus el 49,3 % de casos sin candidemia, sino infecciones urinarias o intraabdominales, entre otras ^24^.

Es importante recordar que, aunque los hemocultivos continúan siendo los pilares para el diagnóstico de la candidemia, su sensibilidad es baja, cerca del 40 al 70 % ^25^. Sin embargo, cuando el resultado es positivo, se puede identificar la especie y, si se solicita, la evaluación de la sensibilidad antifúngica para detectar aislamientos resistentes. En este estudio solo se incluyeron aquellos casos con aislamientos positivos que, por definición, correspondían a una infección por *Candida* spp. confirmada según los criterios del grupo de consenso de la *European Organization for Research and Treatment of Cancer* y del *Mycoses Study Group Education and Research Consortium*^25^ . Sin embargo, es posible que existan otros casos -no incluidos en este estudio- clasificados como candidiasis probable o posible en el consenso mencionado. Sería necesario adelantar otro estudio que incluya todos los pacientes con sospecha de infección por *Candida* spp. para evaluar la sensibilidad de los cultivos realizados.

Otro aspecto por resaltar es el porcentaje alto de aislamientos positivos para *Candida* spp. a partir de muestras del lavado broncoalveolar, pues esto ya indica colonización del hongo. Sin embargo, existen algunas situaciones excepcionales en las que la infección se trata como una candidiasis: cuando hay otro aislamiento positivo para *Candida* spp. en sangre, como en el caso de dos pacientes (uno con coinfección y el otro con segunda infección como se muestra en el cuadro 5); o cuando se sospecha que el paciente tiene neumonía, con una imagen compatible, y sin ningún otro aislamiento que explique la clínica, como ocurrió en otros aislamientos (n = 23).

Se ha documentado que la candidiasis es más frecuente en la población pediátrica hospitalizada que en la adulta ^9^^,^^11^^,^^21^. En este estudio se calculó una incidencia acumulada de 10,6 casos por cada 1.000 pacientes pediátricos admitidos a las unidades de cuidados intensivos pediátricos o la cardiovascular de la institución. Estos hallazgos difieren de los reportados en un estudio multicéntrico realizado por Nucci *et al*. sobre candidemia en la población general de Latinoamérica. En tal estudio, Colombia tuvo la mayor incidencia reportada entre los países evaluados con 1,96 casos por cada 1.000 hospitalizados versus la incidencia general de 1,18 casos por cada 1.000 hospitalizados ^16^. Cabe destacar que dicho estudio multicéntrico no se centró exclusivamente en población pediátrica, mientras que el presente trabajo sí, de hecho, se enfoca en una población muy susceptible: pacientes de una unidad de cuidados intensivos hospitalizados en un centro de alta complejidad y una afluencia elevada de pacientes.

La literatura es congruente en demostrar que la incidencia de candidemia es mayor en los países en desarrollo que en los desarrollados. Por ejemplo, para Argentina y Venezuela se han reportado incidencias de 1,95 y de 1,72 por cada 1.000 hospitalizados, respectivamente ^16^, mientras que para Estados Unidos, la incidencia varía entre 0,3 y 0,9 por cada 1.000 hospitalizados y entre 0,2 y 0,4 en las unidades de cuidados intensivos de Europa ^11^^,^^25^.

La distribución de las especies de *Candida* varía según la región, la institución y la subpoblación. En la cohorte estudiada, la especie más aislada fue *C. albicans* (40 %), seguida por C. parapsilosis (25 %) y *C. tropicalis* (20 %). En su artículo de candidemia en Colombia, Cortés *et al*. reportaron diferentes estudios en los que C. albicans fue la especie predominante en la población general, seguida por *C. tropicalis* y *C. parapsilosis*^18^. De estos resultados se puede concluir que las tres especies mencionadas son las más frecuentes en la población. Sin embargo, el cambio del orden entre las dos especies no albicans puede deberse a que el presente estudio se enfoca en la población pediátrica. Algunos estudios que apoyan esta hipótesis se mencionan a continuación: Jordan *et al*. realizaron un estudio multicéntrico sobre los factores de riesgo y predictores de infección invasora por *Candida* spp. en las unidades de cuidados intensivos pediátricos de España y reportaron que las especies más frecuentes habían sido: *C. albicans* (37,6 %), *C. parapsilosis* (29,6 %) y *C. tropicalis* (15,2 %) ^21^; por otro lado, en el estudio multicéntrico y multinacional de Steinbach *et al*. se reportaron porcentajes similares, ya que *C. albicans* fue la especie más prevalente (44 %), seguida por *C. parapsilosis* (22 %) ^15^; y, en general, esta es la tendencia mundial reportada por los estudios que se centran en pacientes pediátricos ^5^^,^^6^^,^^24^.

Algunos de los factores que podrían contribuir a la alta incidencia de *C. parapsilosis* en la población pediátrica son el uso frecuente de catéteres y otros dispositivos en las unidades de cuidados intensivos pediátricos, pues esta especie tiene una alta afinidad por estos dispositivos y termina adhiriéndose a ellos. Esta adhesión se asocia con la colonización gastrointestinal, más común en niños pequeños, y se ha documentado transmisión horizontal entre las manos de los trabajadores de la salud y los pacientes pediátricos ^26^^-^^28^.

En las últimas décadas, la incidencia de las especies no *albicans* en el mundo ha ido en aumento, como se evidencia en diferentes series y estudios de pediatría ^5^^,^^7^^,^^15^. Los resultados del presente estudio concordaron con lo anterior, pues el 60 % de los aislamientos fueron de especies no albicans. Esta tendencia es de importancia clínica para decidir el tratamiento empírico y evaluar la resistencia a los antifúngicos, especialmente al fluconazol, que es mayor en las especies de *C*. no *albicans*^7^^,^^28^.

Un estudio futuro podría plantear una comparación entre las diferentes especies para evaluar si hay diferencias significativas entre los factores de riesgo o en su prevalencia respecto a otras subpoblaciones. Esto permitiría elaborar recomendaciones más concisas sobre el tratamiento empírico.

En el periodo del estudio se evaluó la resistencia a los antifúngicos en 71 aislamientos. Se resalta que, en ocasiones, se evaluó la resistencia solamente a uno o dos antifúngicos, según los perfiles de resistencia conocidos de la especie aislada. De forma general, las tasas de resistencia son muy bajas, con excepción de los aislamientos de especies como *C. parapsilosis*, *C. lusitaniae* y *C. auris*, que presentaron porcentajes de resistencia altos, por lo menos, a un antifúngico (cuadro 6). Se detectaron dos aislamientos de la especie emergente *C. auris* de un mismo paciente, quien tuvo un desenlace fatal por sepsis causada por este microorganismo. No se observó resistencia en los aislamientos identificados ^16^^,^^29^.

Los factores de riesgo para la candidiasis invasora están bien descritos, entre ellos se encuentran: hospitalización prolongada en una unidad de cuidados intensivos, diagnóstico de neoplasia maligna, uso de antibióticos, catéter venoso central y nutrición parenteral ^9^^,^^12^^,^^21^. Todos los anteriores fueron predominantes en el presente estudio, lo que confirma nuevamente su asociación con el desarrollo de candidiasis invasiva.

En la cohorte evaluada, el 76 % (n = 76) de los pacientes tenía una enfermedad de base, las más frecuentes fueron las cardiopatías en 27 de los pacientes. Esto difiere de lo usualmente reportado en otros estudios, donde la enfermedad más común suele ser oncológica ^9^^,^^12^. En este caso, la mayoría de los pacientes con cardiopatías consultan a la Fundación Valle del Lili porque es un hospital de referencia para cardiopatías congénitas pediátricas, que son atendidas en la unidad de cuidados intensivos pediátricos cardiovasculares.

El uso de antibióticos en las dos semanas anteriores al diagnóstico de candidiasis invasora fue el factor de riesgo más común en el presente estudio. Una media de nueve días (RIC = 6 a 14) de tratamiento antibiótico podría considerarse -según el tipo de infección- como prolongado. Está descrito que el uso prolongado de antibióticos, sobre todo los de amplio espectro, altera la flora gastrointestinal y permite el sobrecrecimiento de especies de *Candida* spp., lo que facilita el desarrollo de candidiasis invasora ^9^. Otro mecanismo que puede alterar la flora gastrointestinal son las intervenciones quirúrgicas abdominales: el 25 % de nuestros pacientes había tenido una cirugía abdominal. Un resultado interesante de la cohorte estudiada fue que solo el 11 % de los pacientes tuvo neutropenia (documentada) en el momento del aislamiento de *Candida* spp., a diferencia de otras series ^9^.

En cuanto a las manifestaciones clínicas de los pacientes con candidiasis invasora, no hay un signo patognomónico para esta entidad, su clínica es inespecífica, por lo que requiere un alto grado de sospecha. La presentación usual de estos casos incluye un cuadro de sepsis o choque séptico, por lo que usualmente se piensa en una etiología bacteriana. Solo cuando hay un fracaso terapéutico con la antibioticoterapia o cuando el paciente presenta factores de riesgo para candidiasis invasora, se sospecha de la misma ^28^.

Por lo anterior, en este estudio se decidió clasificar a los pacientes según los criterios de sepsis pediátrica. La mayoría presentaba choque séptico (30 %) o sepsis grave (30 %) y, en menor proporción, sepsis (10 %) y sin respuesta inflamatoria (7 %). Sin embargo, es preciso resaltar que el 23 % de los pacientes presentaba disfunciones orgánicas sin desarrollar una respuesta inflamatoria sistémica (23 %). Cabe aclarar que el tipo de población en estudio se encuentra críticamente enferma, en su mayoría por causas diferentes a la candidiasis invasora, por lo tanto, la morbimortalidad de estos pacientes es difícil de atribuir únicamente a la candidiasis invasora. Es importante enfatizar el gran reto diagnóstico que representa la candidiasis dadas sus manifestaciones clínicas inespecíficas.

En el presente estudio, el 36 % (n = 36) de los pacientes murió en la misma hospitalización en la que se documentó la candidiasis invasora. Sin embargo, solo 16 casos podrían ser atribuidos a la infección por *Candida* spp., según criterio médico. La mortalidad durante los primeros 30 días después del aislamiento de *Candida* spp. fue del 27 % (n = 27). Las tasas de mortalidad reportadas en los diferentes estudios varían del 7,7 al 26 % dependiendo de la subpoblación estudiada y sus enfermedades de base ^11^^,^^12^.

Según el conocimiento de los autores, este es el estudio con mayor número de pacientes y el más grande realizado en Colombia sobre candidiasis invasiva en una unidad de cuidados intensivos pediátricos. No obstante, su principal limitación radica en su diseño retrospectivo y monocéntrico, aunque fue desarrollado en un centro de alta complejidad (cuarto nivel de atención), que cuenta con una unidad de cuidados intensivos pediátricos y una unidad cardiovascular. Finalmente, es preciso anotar que solo a partir del 2015 se realizan, de forma rutinaria, pruebas de sensibilidad a antifúngicos de los diferentes aislamientos obtenidos.

En conclusión, la candidiasis invasora es una entidad común en las unidades de cuidados intensivos pediátricos y cardiovasculares, y constituye un desafío dada su alta morbilidad, prolongación de la estancia hospitalaria y tasas de mortalidad elevadas. Aunque *C*. *albicans* sigue siendo la especie predominante, las especies de *C*. no *albicans* han mostrado una tendencia creciente, lo que plantea nuevos retos diagnósticos y terapéuticos. La población pediátrica que ingresa a las unidades de cuidados intensivos es particularmente sensible a las infecciones por *Candida* spp. no solo por sus múltiples enfermedades de base, sino también porque es sometida a procedimientos invasores, lo que aumenta significativamente el riesgo de desarrollar candidiasis invasora.
